# Analysis of Sustainable Transport Systems in Service of Selected SEA-EU Consortium Countries’ Airports—A Pilot Case Study of Passenger Choices for Gdańsk Airport

**DOI:** 10.3390/ijerph19020827

**Published:** 2022-01-12

**Authors:** Dariusz Tłoczyński, Agnieszka Szmelter-Jarosz, Sebastian Susmarski

**Affiliations:** 1 Faculty of Economics, University of Gdańsk, Armii Krajowej 119/121, 81-824 Sopot, Poland; agnieszka.szmelter-jarosz@ug.edu.pl; 2 Faculty of Management, University of Gdańsk, Armii Krajowej 101, 81-824 Sopot, Poland; sebastian.susmarski@ug.edu.pl

**Keywords:** sustainable development of transport, SEA-EU consortium, rail airports link, air transport market, sustainable transport, transport demand

## Abstract

The article presents the results of a pilot study, namely a passenger survey on travel choices regarding commuting to the airport in one chosen location (Gdańsk, Poland). The study aimed at establishing which factors which influenced their travel time, assessment of travel time, choosing more or less sustainable transport mode, and also single-mode or multimodal travel. Research results show that choice of the means of transport influences travel time, that the highest travel times are generated by bus and car travel and that assessing the travel time as acceptable or not depends on travel time. However, the longer the travel time, the more likely was the passenger to accept it. What is more, it appeared that a few factors influence choosing a more sustainable transport mode: the purpose of the trip, the start of the trip to the airport, place of living, and job situation.

## 1. Introduction

The years 2020-21 were extremely difficult for global air transport. The crisis caused by the COVID-19 pandemic entailed, first of all, a decrease in air traffic by 76% [1,2,3], contributed to the bankruptcy of several carriers, including AirAsia Japan, Germanwings, Flybe and others [4]. Moreover, global GDP (Gross Domestic Product) fell by 3.4% [5]. In turn, at airports until 2019, the value of passenger service was equal to 3.2% of global GDP [6]. During the pandemic (2020), global airports handled much fewer passengers than the year prior (by 70%). The ICAO (International Civil Aviation Organization) forecast assumes a re-growth of 4.2% on average after a few years [7]; similarly, an increase by 4.0% is believed by representatives of the aircraft manufacturing industry [8,9]. As a consequence of the above considerations, airport traffic should slowly return to the pre-pandemic situation with time. Throughout 2021, a revival of passenger traffic at airports has been visible [1].

To revive the economy, EU Member Countries should focus on the economic recovery strategy and an environmentally friendly path in all sectors of the economy, including transport, in line with the European Green Deal [10] and care about the social dimension of sustainable growth after the pandemic [11]. The policy of European transport sustainable development and national environmental management plans can and will contribute to competitive, sustainable development. The policy of sustainable transport development assumes, among other things, supporting investments in pro-environmental transport [7,12]. This legal framework is a part of environmental dimension of sustainability. The main goals defined by the United Nations [13] relate to numerous social, business and environmental factors [14]. Concern for meeting the needs of future generations is a result of combining these factors with the aim of achieving the goals of sustainable development. Therefore, the need to respect the recommendations of the EU and UN in the context of sustainable development of the transport system results in new requirements for the air transport. One of the transport system elements is the airport links [15].

The guidelines contained in the European Green Policy indicate the need for changes in the delivery of passengers to airports from the unsustainable system (based on car) to rail transport, which has a large capacity, is not exposed to congestion, and above all pollutes the environment to a lesser extent.

One of such activities includes promoting the system of transporting passengers to airports. Airports gather representatives of almost all kinds of means of transport [16]. The projected growth of air traffic along with European policy guidelines will force airports authorities, representatives of central and local government institutions, and transport operators to change the airport links system.

Currently, many European airports have different systems for transporting passengers (by bus, taxi, metro, train, or based on the shared mobility system—carsharing). Therefore, in the context of the presented challenges, it is important to conduct research showing how the airport system works. Following this, we identified the main research problem, showing problems related to the development of the rail delivery and drop-off system and analysing the opinions of passengers travelling to airports.

Although current economic and transport literature delivers knowledge about research in the area of passengers’ commuting to airports, the problem of sustainable airport links system is rarely discussed. Due to the proposed environmental policy of the European Union, there is a need to analyse the system at selected regional airports of the European Union. Therefore, the idea of researching one of the EU airports, Gdańsk Airport (Poland), was proposed. The choice of this airport/country is not accidental. After 2004, Poland had a high passenger growth rate (intra EU—12%, extra EU—40% in 2018/2017) [17], and it is nearly 4% higher than the average for airports located in the EU [18]. Polish airports have not been the object of airport links analysis in the world literature, and Gdańsk is a member of the SEA-EU consortium—European Maritime Universities. The research is meant to be a starting point that will be continued and deepened during detailed research at partner universities.

The implementation of this research based on secondary and primary data will indicate the directions of development of the access and return system at the analysed airports. Furthermore, recommendations for the system’s stakeholders concerning the implemented European Green Deal policy will contribute to benefits for passengers from the functioning of this system. Conducting studies on the issues of a sustainable delivery and return system, based on rail transport is in line with the trends supporting investment processes financed by the European Union. Considering the variety of available transport options to airports and the different attitudes of passengers using these modes of transport, the following research aim has been formulated: to investigate the determinants of passengers’ choice of a particular mode of transportation to and from the airport and to define a model showing the choice of sustainable modes of transport by passengers visiting Gdańsk Airport.

In addition, further research was made, including primary research, of the analysed airports, in cooperation with partners from academic centres of the SEA-EU. SEA-EU is a consortium of universities created as a result of the first edition of the European Commission’s (EC’s) “European Universities” programme. The EC initiative aims to build cross-national alliances of higher education institutions from across the EU with a common long-term strategy and European values. Consortium members are the University of Gdansk (Poland), University of Cadiz (Spain), University of Western Brittany (France), Christian-Albrecht University of Kiel (Germany), University of Split (Croatia) and University of Malta in Valletta (Malta).

## 2. Literature Overview

In the literature related to the management of air transport infrastructure, there are many interesting insights. The vast majority relates to the airport business. In their publications, Doganis, as well as Neufville, Odoni, Janić, Young, Wells and Kazda, Caves [19,20,21,22,23] focus on airports as a business entity. They point out aspects of revenue, cost, management, planning of airside (the part of an airport closest to an aircraft, which is bordered by security screening) and landside (accessible part of the airport up to the security check boundary), environment, capacity, safety of specific infrastructure elements, as well as the role of airports in terms of economic, social, and operational factors, i.e., handling services. However, there is a lack of current research on commuting efficiency, capacity, and management between the city centre and the airport. According to Ashford et al. [24], an analysis of the airport access problem was carried out in the branch system, indicating a wide choice of cars by passengers using airports, but the remote period of the presented research must be taken into account. In addition, they analyse the availability of different modes of transport concerning the time-of-flight operations (slots). Their study takes into account the typically operational nature of airport operations.

It should be noted that the airport is an integration node for all modes of transport [25] and has a key role in the economic development of regions [26]. The studies presented here are based on the planning of the airport area and the benefits of operation for stakeholders without analysing the demand for airport links. For example, [27] concludes that transport between city centres and airports can be more burdensome for passengers in terms of expenditure, travel time and baggage handling activities than air transport. It is most probable that cities with an airport shuttle system based on rail transport have higher productivity than cities without such a system. This justifies the purposefulness of railway investments between airports and city centers. It is important to agree with this research [27], in the sense that investment in an airport rail system will be a key element of a region’s competitive advantage, as well as the development of regions. Although the authors indicate that the research conclusions can be applied to the economic potential of regions of Asia or Middle East, such solutions may also be implied for America or Europe.

Research on factors influencing the choice of airport links system is shown in [28]. The Big Five Personality Factors were explored, i.e., personality, attitudes, identity, perception and feeling, the analysis of which showed the existence of several market segments which are sensitive to price, convenience, and time. An interesting methodology was implemented in this study, first collecting data based on a questionnaire and then analysing it based on Hybrid Discrete Choice (HDC) model. The time involved in reaching the airport is the research subject in [29]. The authors analysed the costs of the variability of the travel time of air transport passengers. This is another point of research into the time and cost of airport accessibility as a value to the passenger. For travelers, reliability is essential as the costs of delaying a flight are very high. At the same time, the authors indicate the best time to travel to the airport and what costs passengers may incur in connection with the travel to the airport. Additionally, the high costs of being late and the change in the way of getting to the airport from a car to public transport are presented in [30].

Choosing how to travel to the airport as a result of individual behaviour was the subject of research by Choo et al. [31]. They performed descriptive analyses to investigate the relationship between travel purpose, travel time and cost, and the availability of different modes of transport to airports. At the same time, the team developed a regression model for multimodal transport in airport commuting. The results indicate a need to divide passengers according to the purpose of travel and adapt the transport system to them. This approach to research shows the complexity of the research problem. The logit model presented significant results, and this model is widely used in this type of research.

A different approach to the problem of transporting passengers to and from the airport is related to the segmentation of passengers using air transport. The necessity of segmenting airport links transport is a consequence of the preferences and behaviours of people who pursue various variants of air travel, taking into account the type of travel: domestic or international or the travel class. The choice of the means of transport is determined by many factors, including the distance between the city/cities and airport, travel time by modes of transport, number of passengers operating at the airport, specificity of air traffic, involvement of the city, regions, and transport companies in the sustainable development systems. Moreover, Budd et al. indicate the critical variables influencing the choice of transport mode: the location of the airport, its distance from the city centre, the region, the local topography, the specificity of the airport passenger service, the possibility of other alternative delivery systems, as well as cooperation with local and regional authorities [32]. At the same time, this team points to the need to reconcile business interests with the issues of sustainable transport development. This approach to sustainable development of transport points to a potential direction for action by public institutions, e.g., the EU.

Another factor influencing the choice of means of transport in the airport —city centre route is ecology [33]. The authors point to pro-ecological attitudes related to the choice of rail transport for going to the airport, which in the current environment is a priority for policymakers.

In addition to the environmental factor, it is necessary to indicate the important factors for investments which relate to the construction of the rail airport link. Coogan points to other supply-side aspects, which of course influence the later choice of sustainable modes of transportation to the airport (proportion of air travellers with trip ends in the city or for whom the city is a transit point, characteristics of passengers using air transport, time of regional travel, local means of transport, availability of railway stops and difficulties related to overcoming architectural obstacles, accessibility of the railway network, flexibility of train arrivals and departures for air traffic, frequency of rail services [34].

Passenger expectations regarding rail transport services vary. Rail airport link providers offer differentiated services, e.g., faster travelfrom the city centre to airport at a higher price (Heathrow Express) or longer journey at a lower price. Moreover, in cooperation with railway operators, some air carriers offer a special ticket and luggage package (Lufthansa Express Rail) [35]. This is an example of sharing responsibility for the environmental impact of business.

From the regulatory side of the market—public institutions are emerging guidelines that seek to change travel behaviour in choosing to travel to the airport.

The European Union defines priorities in the field of promoting sustainable transport. However, the activities of all EU institutions are not limited only to supporting transport in the last few years, but have a much broader dimension, as the entire air transport sector is considered one of the leading polluters and environmental threat factors. Many publications [36,37,38] show that air transport emits 3% of global CO_2_, hence the guidelines [39,40] of the European authorities aimed at reducing the negative impact of air transport on the environment, at the same time indicating that cooperation and integration with rail transport will induce the expected results for creating sustainable transport system [41].

National transport policies are consistent with the EU transport policy and constitute its integral part, assuming the reduction in natural pollution due to the implementation of the European Green Deal, the Paris Agreement or the strategy for Sustainable and Intelligent Mobility [10,42,43] for the implementation of business. For example, one of the instruments includes investing in and promoting rail airports link.

Summing up, the sustainable transport system to airports is currently perceived not as an alternative to the car, shuttle bus or bus connections, but as a necessity for implementing national and European transport policies.

As a result of the literature analysis, in the current paper the authors represent different approaches to choosing an airport travel system. Three main areas of research dominate: analysis and modelling of sustainable travel behaviour, classification of the most important factors influencing public transport choices, particularly the valuation of time as a cost and the cost of transport, and the shaping and planning of the supply of airport links. All of these aspects are equally important to developing a sustainable airport travel system, which is particularly relevant in terms of EU regulations and policies.

## 3. Materials and Methods

### 3.1. Research Framework

Based on the literature on the subject, it was found that airports will conduct many activities related to the development of sustainable mobility in the near future. The first element of the research was a review of the literature on the functioning of airports and legal acts related to implementing the policy of sustainable transport development in Europe. On the basis of the literature review, the factors determining the behaviour of passengers during the selection of the delivery and return system were determined. The research approach was similar to that made by Choo, You and Lee [31]. Ultimately, the analysis would cover all airports located in countries—members of the SEA-EU scientific consortium [44]. The airports in Gdańsk, Hannover, Malaga, Bordeaux, Zadar and Valletta [45,46,47,48,49,50] were initially analysed, suggesting the implementation of railway transport investments are sensible for supporting the transport system in those locations. The final result of the research will be a comprehensive study among all airports in countries where SEA-EU consortium research centres are located. To this end, an initiative will be launched that employees of universities including University of Cadiz, University of the West in Brittany, Christina-Albrecht University in Kiel, University of Split and University of Malta in Valletta, and University of Gdańsk conduct research in the mentioned airports.

This paper presents the results only of the first phase of the more extensive research project, namely a case study for Gdańsk Airport (including railway system) [51]. To achieve the research goal stated in the Introduction, a survey among airlines passengers was conducted on a sample of 286 respondents, randomly selected in the public area of the airport, who were flying by regular air carriers. The research was carried out in the third week of June, in the run-up to the pandemic (see also Section 3.3). Therefore, to sum up, the research findings this paper presents are based on a case study of Gdańsk Airport (see Section 3.2), prepared by conducting a survey among passengers (see Section 3.3), which results were analysed with the use of statistical analysis (see Section 3.4 and Section 4).

### 3.2. Case Study

**Figure 1:**
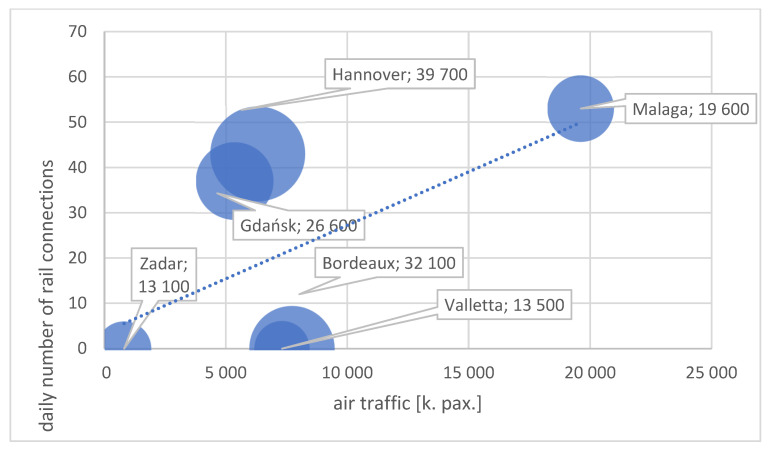
The relationship between the daily number of rail connections and air traffic/the region’s economic potential. Explanations: circle (airport) size—economic potential of the region (GDP per capita [EUR]); pax.—number of passengers. Source: own elaboration based on [45,46,47,48,49,50].

The analysis of the mentioned cities having airports within the SEA-EU consortium provided information about the possibilities of reaching the airport using different modes of transport. It was discovered that every city has a specific transport and logistics system, enabling the use of particular transport modes to get to the airport. In fact, the richest possibilities were recognized in Gdańsk (all of the transport modes except trolleybuses and trams). Additionally, in the light of the goals of this paper, only those cases where implementing sustainable transport is possible were analysed. Therefore, three destinations were identified: Malaga/Costa del Sol, Hannover and Gdańsk, each having an excellent railway link—a railway line straight to the airport from many locations within their regions (see Figure 1). This enables the creation and maintenance of sustainable mobility patterns of passengers coming to the airport. This led to the choice of the case study method to provide initial insights and initial research results to optimize and improve the research approach to be implemented in the whole analysis. Therefore, Gdańsk Airport was chosen as the location to be examined in the area of the willingness of passengers to use particular means of transport to get to the airport to assess their sustainable transport attitudes.

In economics and management, and more generally—in social sciences, a case study is a popular research method [52,53], providing interesting results helping to build theory and to plan and design further studies [54]. It was often implemented in transport-related research, especially when examining passenger behaviour [55,56,57]. It was successfully used in the area of sustainable transport [58,59,60,61]. Even if it is well-known, there are still different approaches to its design, research procedure steps, and theory building [62]. In this study, the empirical evidence, proven by obtained survey data, was helpful for building a midrange theory (theory propositions to be verified on the biggest group of airports) [63]. It was an excellent method to consider individual characteristics of the region and the airport itself. While designing the method implementation steps, it needed to meet the requirements stated in the case study-related literature: to build it in a rigorous, robust way to include practical evidence in theory building [64].

According to Yin [51], the research procedure should answer the research questions, which in this paper are as follows:

- RQ1: If the commuting to the airport lasts longer, does it impact on traveller’s satisfaction?
- RQ2: What are the factors influencing using more sustainable transport modes by passengers?

Despite some unreasoned critiques about the case study as a non-reliable method, it can be and should be designed as a rigorous and robust procedure to use the case study to build some theory to be verified in further studies. It is known as well for its strong practical evidence [52,65,66]. In this study, the case study was planned on the basis of [51,52,53,63] and included the following steps:

1. Phase of design:
  a. Identification of research problem and research questions (Section 1, Section 2, Section 3.1 and Section 3.2);
  b. Defining the method to collect data (Section 3.2 and Section 3.3).
2. Phase of carrying out the survey:
  a. Design, validation and correcting the questionnaire (Section 3.3);
  b. Collecting data from survey (Section 3.3).
3. Phase of data analysis and concluding the results
  a. Choose the statistical methods for data analysis (Section 3.4);
  b. Preparing data analysis report (Section 4);
  c. Comparing with other theories (Section 5);
  d. Concluding the cross-case report (Section 6).

### 3.3. Survey Method

To achieve the research goal, a survey for airline passengers starting their trip in Gdańsk was prepared. The population of passengers of the Gdańsk Airport counted 1.71 million passengers in 2020 and almost 5.4 million passengers in 2019 [67]. Since the survey was collected just before the pandemic, the number of 5.4 million was taken as a basis for calculating the sample size. The fraction was uncertain since no one studied how many of the passengers used some mode of transport to commute to the airport. The fraction is the presence of the feature in the studied population. In this case, it is the share of people commuting to the airport in the number of all passengers using the airports. However, the airport is located outside the main city area, so every passenger had to use some means of transport to get there. From the other point of view, there are many ways to get to the airport: own car, taxi, carsharing, micromobility—bike, motorbike, scooter or moped—own or shared, regional rail and bus [28,68]. Therefore, assuming the highest possible fraction of 0.9, the confidence level as 95% and maximal error of 5%, the sample size should be 138. If the confidence level would be 99%, the sample size should be 239 [69,70,71,72]. Therefore, it was decided that at least 239 questionnaires should be completed and this minimal number of observation was reached and exceeded—finally, the survey was held in a group of 286 randomly chosen passengers. Trained surveyors carried out the survey. The questionnaire contained 16 questions in three topical groups:

(1) Flight characteristics:
  - Flight with transfers (yes/no);
  - Final destination;
  - Type of flight;
  - Type of service provider;
  - Class;
  - Purpose of journey.
(2) Transport to the airport:
  - Start of travel to the airport;
  - Means of transport;
  - Time of travel;
  - Assessing the travel duration (acceptable or not).
(3) Socio-economic characteristics of passenger:
  - Job status;
  - Place of living (city name and size, country);
  - Nationality;
  - Age group;
  - Gender;
  - Frequency of flying.

Table 1 presents descriptive statistics for the surveyed group (excluding the destination of travel since many possible answers, over 50 in the surveyed group). For each potentially significant variable for further analysis (Table 1), the type of the variable and all of the possible values were indicated to adjust the statistical analysis. What is visible from the obtained data is that most of the respondents lived in Poland and were Polish. The most of surveyed passengers were of working age (over 75% of all respondents) and male (over 60%). Almost 1/3 were managers or directors and many of them were using air transport more frequently than once in two months (over 32%).

In the matter of the current flight that they were waiting for during the survey, in most cases passengers were waiting for a continental flight (3 of every 4 flights), and in almost 60% of cases, it was a flight without transfers. The surveyed group was divided almost into half for the subgroup travelling with low-cost transport service providers and legacy transport providers. Usually, the flight offered no class division, and if offered, the passenger usually chose the economy class. The most popular travel purposes were business and touristic (in every case, over 30% of respondents and in over 85% of cases, it was a short-term travel).

The third subgroup of variables were those related to the trip from the starting point to the airport. Almost 63% of the respondents were commuting from the Tricity agglomeration (Gdańsk, Sopot, Gdynia) or metropolitan area (Reda, Rumia, Wejherowo), including almost a half commuting from Gdańsk, so they had to travel 15 km or less to the airport. In the case of the agglomeration or the metropolitan area, it could be more than 15 and less than 50 km. Almost 10% started from the Pomeranian Region (voivodeship) outside the agglomeration or metropolitan area, so they travelled from over 15 km to a maximum of 240 km. Almost 25% of respondents were travelling from starting point outside the region, so they had to travel at least 70 km. In fact, those groups could use almost every transport mode to get to the airport. Using a taxi was only sensible for travellers from Gdańsk or agglomeration or some chosen parts of the region. Finally, 4.2% of respondents were from abroad, but in most cases from the Kaliningrad Region, so they had to carry out a journey of a minimum of 130 km and a maximum of ca. 350 km. For them, car or rail would be the best choice relating to mode of transport.

**Figure 2:**
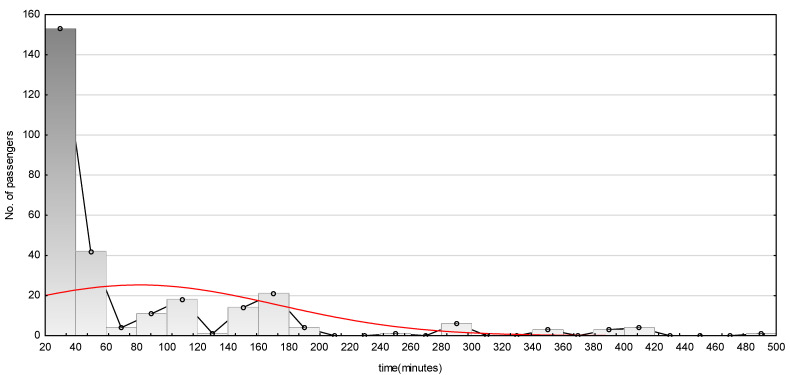
Distribution of observation for the time of travel. Explanation: red line represents normal distribution of data.

Importantly, almost 75% of the surveyed passengers admitted that commuting time was acceptable and for only 3.5% was it definitely too long. As indicated in Table 2, the respondents travelled at least 20 min (minimum, see Figure 2) to the airport from their starting point and a maximum of over 8 h. Looking at the descriptive statistics, it is clear that the dominant (mode) value was 20 min and half of the respondents travelled 40 min or less (median). This suggests that the point of starting the journey was located nearby the airport (Tricity agglomeration or communes close to the airport). The next 25% travelled between 40 and 120 min (Q3). This can be assigned to a distance of more than 40–50 km and no more than 120 km; therefore, the journey probably started outside the agglomeration. However, the travel time was very diverse since the var. ratio is very high. Usually, when this ratio is 25–30% or less, it is interpreted as low; when 30–50% it is interpreted as a medium; when 50–100%, it is interpreted as high; when above 100% it is interpreted as very high [73]. In this case, the standard deviation is 110.47% of the mean travel time, so the differentiation of obtained results is very high, this means that the surveyed group was very diverse, which is good for the reliability of results.

### 3.4. Statistical Analysis

Building a proper set of different analyses and tests was essential to achieve the research goal. After designing the survey questionnaire, it became evident that there should be more than one analysis to show the significant factors affecting passengers’ choices.

Firstly, we decided to look at the relations between the purposes of trips and choosing the mode of transport (multimodal or single mode, see Section 4.1). Then, we aimed to check if travel time depends on the chosen means of transport (see Section 4.2). For that purpose, taking into consideration the nature of dependent (continuous, quantitative) and independent (nominal, qualitative) variables, we decided to use the non-parametric test—ANOVA Kruskal Wallis test (made in STATISTICA software by StatSoft Polska, Kraków, Poland) used as well in similar studies [74,75] (see Section 4.3). Thirdly, it was essential to check if the assessment of the trip duration (acceptable, rather too long or definitely too long) depends on the travel time in minutes (see Section 4.2). Since the assessment was subjective and described by only a few options to be chosen by the respondents, it was not directly measurable. In this regard, the best choice was multinomial logit or probit, as well as looking at similar research [76,77]. Therefore, the logit function was chosen. Finally, we needed to assess what factors influence the choice of more sustainable transport modes. For that purpose, the dependent variable was created assuming that using a car or taxi—an individual motorized form of transport—is the least sustainable, using motorized public transport (bus) is moderately sustainable and using the other modes (rail or multimodal) would be the most desirable from the point of view of sustainability. Then, the nature of this variable (most sustainable vs. moderately sustainable vs. non-sustainable as dependent variable) determined the nature of the proposed model. Considering the group of potentially significant independent variables from Table 1, the ordered logit model was the most suitable to prepare and test. Ordered logit was used to assess the importance and significance of different factors in many research in transport, including passenger behaviour [78,79] and sustainable transport [80]. The model was first estimated for all of the proposed variables to see which of them were significant and which not. Then, according to good practices in statistics, insignificant variables with the highest *p*-value from the *t*-test were eliminated step by step, until the model contained only variables significant at level 0.1 (see Section 4.2 and Section 4.3). Moreover, the metrics for the model were checked at every stage of variable elimination. Those metrics were: Akaike Information Criterion (AIC), Bayesian Information Criterion (BIC), the share of correct prediction cases, McFadden R-square and corrected R-square (see Table 3), correlation matrix and maximum likelihood test type 1 (see Appendix A, Table A2). The best model should have the lowest AIC and BIC from all calculated models. It was clearly stated that the final model met all the formal requirements and could be interpreted to provide research insights.

## 4. Results

### 4.1. Cross-Analysis of the Purpose of Travel and Use of Transport Modes

Most of the passengers travelling for business purposes used a car or taxi. They were the biggest group of taxi users. However, this group is not the least sustainable among the surveyed 286 persons because they only comprise almost 1/3 of car users (see Table 4). Tourists are the least sustainable because usually use the car (almost half of car users). Nevertheless, they are also the biggest group of bus and agglomeration rail users. Naturally, they are the only group of plane users, but this is an obvious result (they were in the middle of their trip when transferring from one plane to the other). Respondents visiting family usually used a car to get to the airport (almost 60% of them used this transport mode). The second chosen mode was for them was the agglomeration fast rail. They usually do not use the bus or taxi and combine transport modes to get to the airport. People travelling for long-term work purposes usually use cars or fast rail, but they represent one of the most prominent groups with multimodal transport choices. Probably because of the trip’s goal, they cannot use their own means of transport since they are leaving the country for a longer time.

The other findings resulting from Table 4 are as follows:

- the less sustainable group of travellers are people travelling for business purposes (over 74% using individual motorized transport) and visiting family (over 67%);
- the most sustainable group of travellers are people travelling for work purposes (over 45% of them using fast rail, bus or combine different modes) and tourists (almost 39% of them);
- most multimodal travellers are tourists (almost one-third of all respondents declare multimodal transport choices).

### 4.2. Time vs. Use of Means of Transport

The next step in the analysis was to check if the time of travel from starting point to the airport depended on the transport mode or not (see Section 3.4). Since the distribution of the travel time was abnormal (Chi-sq = 373.66, *p* = 0.000), the ANOVA Kruskal-Wallis test was held with the following statistical hypotheses:

**Hypothesis** **1** **(H1).** *Using different means of transport with the same time of travel*.

**Hypothesis** **2** **(H2).** *Using different means of transport with different time of travel*.

**Figure 3:**
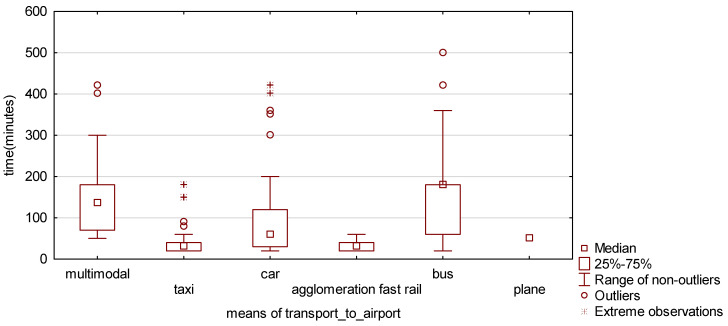
Box-whisker test for travel time according to the chosen means of transport for travelling to the airport.

In the box-whisker chart (see Figure 3), it is visible that the time of travel is different for various transport modes. For fast rail and taxi it is shorter and for the bus or multimodal travels it is longer. To be sure, it was tested using the ANOVA Kruskal-Wallis, for which the statistic was 100,19 (df = 5, *n* = 286) and *p*-value was 0.000. As always, when *p*-value in the test is lower than the significance level, usually 0.05, the null hypothesis is rejected in favour of the alternative hypothesis. Therefore, it was confirmed here that travel time depended on the chosen transport mode. The median test confirmed that result (Chi-square = 97.76, *p*-value 0.000).

The duration of the trip could have an impact on the perception of perceiving of the transport mode by travellers. Therefore, we checked if the assessment in this area depended on the travel time and other variables. The dependent variable could have three values: acceptable, rather too long or definitely too long. The ordered logit model was prepared, and it was clear that the assessment of tip time depended on a few factors (see Table 5 and Table 6 and Appendix A, Table A1). Again, when the *p*-value was lower than 0.05, the variable was defined as significant.

For assessing the time of travel impacted only a few potentially important variables:

- if the travel was for a business trip, then the passenger usually did not complain about the trip to the airport being too long (they usually used taxi or car, so the transport modes with a shorter average time of travel than other modes);
- if the traveller was a regular employee (not a business owner nor manager or director), this person assessed, on average, time of travel as more acceptable;
- if the travel time was higher, the lower the chance was of it being assessed as too long (probably people having a long distance to the airport were prepared for a long trip, so they were not surprised by its duration and complained less);
- if the traveller was female, on average, they complained less about travel time being too long;
- passengers starting their trip to the airport outside the agglomeration or metropolitan area assessed the travel time as more acceptable in comparison to other passengers groups (again, they could be prepared that the trip will last longer, and people from the agglomeration wanted to reach the airport very quick).

### 4.3. Choice of Sustainable Transport Mode(s)

The last of the prepared models was oriented to check which factors affect the sustainability of passengers’ trips. After a few iterations with eliminating the independent variables, the final model was estimated (see Table 7 and Table 8, Appendix A, Table A2; again, a *p*-value lower than 0.05 determined the significant variables in the estimated model).

The model provided many important insights for showing the sustainability of travel choices of airlines passengers in Gdańsk Airport, namely:

- passengers representing the widely understood management, pensioners and unemployed were more likely to choose sustainable transport modes than persons on the other positions (e.g., regular employees);
- people assessing the time travel as rather too long or definitely too long were more likely to choose less sustainable transport modes (since they are probably more willing to use car or taxi);
- people choosing the services of low-cost airlines are less willing to choose sustainable modes;
- passengers living outside Poland were more willing to use sustainable modes of transport;
- people starting their trip to the airport from the Pomeranian Region were more willing to use sustainable modes of transport;
- people starting their trip to the airport from the city of Gdańsk were less willing to use sustainable modes of transport (since they mainly used a car or taxi because of the average shorter distance than the rest of the respondents);
- for people starting their trip from outside the region or from agglomeration, the use of more or less sustainable modes of transport was not estimated as significant.

## 5. Discussion

Considering the relationship between trip purpose and mode choice, it is important to establish the source of the significant advantages of a car over rail or bus in business-related travel. The car is less preferred compared to modes of transports in the group of residents, including those living near an airport and those in a metropolitan area. The authors believe this may be due to several specific reasons:

- convenience and economic and accounting aspects. Business travellers are usually perceived to be less price-sensitive in their choice of means of transport, treating the transfer by taxi and car as an element of prestige (travelling alone and comfort) [81]. At the same time, it is worth emphasizing that transport expenses are tax-deductible for the company following tax regulations. It is worth stressing that the acceptability of taxi and car transportation costs by travellers could be the subject of further research. This will change because e.g., rising fuel prices, directly translating into costs. However, in this study it appeared that they are more willing to be sustainable—and this can be related to their generally higher level of education and environmental awareness [82,83];
- specifics of the Tricity agglomeration. The Tricity agglomeration stretches along the coast of the Baltic Sea. On one side it is limited by water, on the other by forests [74]. The agglomeration cities are connected by a single main artery, equipped with bus lanes and a railroad line. Taking advantage of this habit, the local authorities of Pomeranian Voivodeship have implemented the largest railroad investment project after World War II in Poland, which includes the railroad connection of the airport with the agglomeration [84]. This should be seen as a large factor in shaping the sustainable transport behaviour when reaching the airport (this is clearly noticeable in the case of people starting their journey to the airport from the region).

The need for further education on sustainable transport is clear from the research. People living abroad use a sustainable transport system more often than those living in Poland; this may result from a greater responsibility for the environment and the offer of more or less sustainable airport links.

The authors also note an apparent disconnection between the jobs of respondents and the transport modes used. Non-workers and retirees are more likely to use sustainable transport to get to the airport than people who are representatives of companies, generally confirming the fact of different travel choices according to life stage [85,86,87]. The problem of lower willingness to use more sustainable travel modes among the people in working age can be solved in two ways:

- through education and shaping of pro-ecological attitudes—in this case, future benefits in the form of conscious use of sustainable transport should be emphasized;
- by adding appropriate prestige to sustainable means of transport (which requires investment)—in this case, changes can be made in the choices of business travellers—they will use sustainable transport to a greater extent.

It is also interesting to note the strong correlation between economic development and the volume of air traffic. Such a relationship indicates not only that the supply of air transport services should be developed, but from the point of view of demand, the accessibility of the feeder system should be increased. Of course, at the relatively small airport of Valletta in Malta, the scale of investment in sustainable transport will be negligible compared to the economic potential of airports in Malaga, Hannover or Bourdeaux. EU guidelines indicating the promotion of sustainable transport will, among other things, contribute to the reduction in environmental pollution generated by taxis and car.

Distinct from Choo [24], who focused on domestic airports, the authors here point out the need to extend the study to airports in SEA-EU countries. The next stage will be to conduct a qualitative assessment of the airport links system at the analysed airports, in collaboration with the research centres forming the SEA-EU consortium. Such research will provide the information for possible directions of development.

## 6. Conclusions

Summarizing the results of the authors’ research, it is important to note the contribution of the article to the theory and policy. The results of the conducted research made it possible to answer the research questions posed and achieve the research goal. They show that the choice of the means of transport on the route airport/city centre is influenced by the time and type of travel, the type of flight or airline, as well as the place of residence, social status, and stage of life. This is not a new result in the literature but indicates that the same factors determine mode choice at the small regional airport (Gdańsk Airport, 5.4 million passengers in 2019) as is the case of airports/cities with high economic potential (literature review studies usually refer to airports in South Korea, US, UK or big EU cities). Previous studies have not analysed airports located in Central Europe (including Poland), where air traffic growth has been higher than the EU average dynamic. Therefore, to some extent, the research gap was filled by this very paper.

The authors obviously, as a result of the analysis of the literature review, note that the conducted research does not take into account time as a cost of travel to the airport, nor does it take into account the impact of weather conditions and the amount of baggage, all of which can be the subject of further in-depth research in this area. However, the authors point out the need for a holistic approach to sustainable transportation. Similarly to Choo [24], they emphasize the need to include the stage of getting to the airport and returning from the airport in the concept of the transport stream. However, they take a different approach to the notion of sustainable transport in the context of arrival and return by looking at it from the perspective of efficiency, safety, and environmental friendliness, elements that are important from the point of view of the European Green Deal. The research results clearly show that the use of sustainable transport depends on the place of residence and education/experience.

Undoubtedly, the choice is influenced by the location of the airport and the frequency of operation of the airport links system, which is highlighted in several items in the literature. According to the research, the inhabitants of regions, where in addition to road connections, there are also rail connections, use sustainable transport to a greater extent. Indeed, such a choice is a factor of social awareness, resulting from responsibility for ecology. The same is true for education/experience.

The high success of rail transport in getting passengers to and from airports among pensioners and the unemployed is due to economic factors, but when considering the management/employees relationship in terms of choice of transport, awareness of the concept of sustainable transport is beginning to play a more significant role. This is indicated among other things by the fact that the use of rail transport is more expensive than travelling by bus, but the journey takes less time. Similar conclusions were reached by authors dealing with the issue of mode choice during their literature analysis. For executives, the issue of cost and travel time may be of secondary importance, but not for business trips. Only passengers travelling as tourists, visiting family, or working are more likely to choose the most efficient and sustainable way to reach their destination than passengers travelling for business purposes. This is due to different needs. In this study, passengers travelling from other distant regions choose a car—this is far from the suggestions in the literature. This in connected to poor accessibility to public (rail) transport, especially High-Speed Railway (HSR). This indicates the potential directions of development of the railway system in Poland.

In summary, the authors recognize the need to expand the research noting that the research is only a case study of one city, which is a main limitation of the article. However, this is only the initial phase of a larger research project, so our future research will focus on seeing whether or not the same factors influence mode choice in other locations in the SEA-EU region. Therefore, future research will be focused on deepening the research for other airport locations.

At the same time, the authors will also consider more factors influencing travel choices in the future. However, they realise, as highlighted in the literature review, that unfortunately, it is impossible to analyse all possible factors due to their unlimited number, and the size of the questionnaire. Therefore, the next step will also be to build a model of the relationship between the choice of sustainable transport to the airport and the specifics of air traffic (low-cost carriers/legacy carriers/charter or domestic/international-intra/extra EU) at selected airports.

The research conducted by the authors is also of practical importance. It can be an element of an in-depth analysis of airport stakeholders and, in perspective, the subject of allocation of funds for investment in peri-airport infrastructure by both airports and government or local authorities responsible for transport infrastructure in the context of airport access. Understanding the attitude of passengers to the cost of service, travel time, awareness of the concept of sustainable transport, place of residence, and nature of travel, one can more effectively diversify the transport network and manage it more efficiently. As a result, the results of the authors’ research can be used both at the level of operational management of the existing transport network, as well as directions for investment outlays for its improvement or diversification, i.e., adjusting the transport offer to the growth of air traffic. The authors point out that airport managers must be aware that with the increase in air traffic at airports, congestion in the airport area, there will be an increasing demand for a sustainable passenger transport system to airport terminals. Moreover, operators will also be forced to make place for more passengers using a private car, in case of more luggage or bad weather conditions, as pointed out in the literature.

The authors propose an innovative approach in the form of involvement of partner universities associated in the SEA-EU consortium in the research process. Consequently, the authors aim to create a forum for the exchange of experience (using SEA-EU consortium) concerning sustainable air transport understood in a holistic way.

## Figures and Tables

**Table 1:** Descriptive statistics for the surveyed group.

Category	Variable	Values	Share in %	Type of Variable	Category	Variable	Values	Share in %	Type of Variable
Flight characteristics	Type of flight	Continental (letter C)	77.97	Qualitative	Profile of traveller	Place of living	Other	33.57	Qualitative
		Domestic (D)	10.84				Poland	66.43	
		Longhaul (L)	11.19			Nationality	Polish	83.92	Qualitative
	Flight with transfer	No	59.09	Binary			Other	16.08	
		Yes	40.91			Age group	20 or less	3.15	Ordinal
	Type of provider	Low-cost (LCC)	50.35	Qualitative			21–30	28.32	
		Legacy (LT)	49.65				31–40	32.17	
	Class	Without class (0)	60.84	Ordinal			41–50	24.83	
		Economy (1)	33.22				51–60	6.64	
		Business/first (2)	5.94				61–70	3.85	
	Purpose of travel	Business	35.66	Binary			Above 70	1.05	
		Touristic	31.47	Binary		Gender	Female	39.16	Qualitative
		Visiting family	18.18	Binary			Male	60.84	
		Work (for longer time)	14.69	Binary		Position	Executive/director	10.49	Binary
Transport to the airport	Start of travel to airport	Poland (other locations; P)	23.78	Qualitative			Manager	21.33	Binary
		City of Gdańsk (M)	48.60				Specialist	17.83	Binary
		Tricity agglomeration (A)	14.34				Own business owner	11.54	Binary
		Pomeranian Region (R)	9.09				Regular employee	20.28	Binary
		Outside Poland (Z)	4.20				Student	10.84	Binary
	Means of transport to airport	Multimodal	6.29	Qualitative			Not employed	3.15	Binary
		Taxi	18.88				Pensioner	2.45	Binary
		Car	46.85				Other	2.10	Binary
		Agglomeration fast rail	18.88			Frequency of flights (last 12 months)	Zero	15.03	Ordinal
		Bus	8.74				1–2 times	26.57	
		Plane	0.35				3–5 times	26.57	
	Assessment of time to get to airport	Acceptable (1)	74.13	Ordinal			6–10 times	14.69	
		Rather too long (2)	22.38				Over 10 times	17.13	
		Definitely too long (3)	3.50						

**Table 2:** Basic statistics for travel time.

	Mean	Median	Mode	Minimum	Maximum	Q1	Q3	St. Dev.	Var. Ratio
Time (in minutes)	81.68	40	20	20	500	30	120	90.23	110.47

**Table 3:** Metrics for the ordered logit model presented in Section 4.2.

Metric	Df	Stat.	Stat/Df
Deviation	558	395.916	0.709
Scaled deviation	558	395.916	0.709
Pearson’s Chi-square	558	505.365	0.906
Scaled Pearson’s Chi-square	558	505.365	0.906
AIC		423.916	
BIC		475.100	
Log(likelihood ratio)		−197.958	

**Table 4:** Crosstable for purposes of the trip and chosen modes of transport.

Category		Multimodal	Car	Taxi	Agglomeration Fast Rail	Bus	Plane	Total
usiness	Number of cases	3	33	43	15	8	0	102
	% in column	16.67%	61.11%	32.09%	27.78%	32.00%	0.00%	
	% in row	2.94%	32.35%	42.16%	14.71%	7.84%	0.00%	
	% in total	1.05%	11.54%	15.03%	5.24%	2.80%	0.00%	35.66%
Touristic	Number of cases	6	13	41	17	12	1	90
	% in column	33.33%	24.07%	30.60%	31.48%	48.00%	100.00%	
	% in row	6.67%	14.44%	45.56%	18.89%	13.33%	1.11%	
	% in total	2.10%	4.55%	14.34%	5.94%	4.20%	0.35%	31.47%
Visiting family	Number of cases	4	4	31	10	3	0	52
	% in column	22.22%	7.41%	23.13%	18.52%	12.00%	0.00%	
	% in row	7.69%	7.69%	59.62%	19.23%	5.77%	0.00%	
	% in total	1.40%	1.40%	10.84%	3.50%	1.05%	0.00%	18.18%
Work (for longer time)	Number of cases	5	4	19	12	2	0	42
	% in column	27.78%	7.41%	14.18%	22.22%	8.00%	0.00%	
	% in row	11.90%	9.52%	45.24%	28.57%	4.76%	0.00%	
	% in total	1.75%	1.40%	6.64%	4.20%	0.70%	0.00%	14.69%
Total	Number of cases	18	54	134	54	25	1	286
	% in total	6.29%	18.88%	46.85%	18.88%	8.74%	0.35%	

**Table 5:** Results for ordered logit for assessing the trip duration.

Category	Level	Coeff	St. Err/	Wald Stat.	Upper 95%	Lower 95%	*p*
Const1		2.196	0.403	29.698	1.406	2.986	0.000
Const2		5.006	0.588	72.441	3.853	6.158	0.000
business		−0.793	0.348	5.178	−1.475	−0.110	0.023
regular employee		−0.599	0.372	2.586	−1.328	0.131	0.108
time (minutes)		−0.008	0.002	11.819	−0.013	−0.004	0.001
gender	female	−0.364	0.155	5.521	−0.669	−0.060	0.019
start_travel_to_airport	*P* *	−0.673	0.276	5.938	−1.215	−0.132	0.015
start_travel_to_airport	M	0.741	0.382	3.759	−0.008	1.490	0.053
start_travel_to_airport	A	0.028	0.412	0.005	−0.780	0.836	0.946
start_travel_to_airport	R	−0.846	0.365	5.355	−1.562	−0.129	0.021
Scale		1.000	0.000		1.000	1.000	

* M—Gdańsk, A—Tricity Agglomeration, R—Pomeranian Region, *P*—outside the Pomeranian Region.

**Table 6:** General results for ordered logit from Table 5.

Category	Wald Stat.	*p*
Const	76.893	0.000
business	5.178	0.023
regular employee	2.586	0.108
time (minutes)	11.819	0.001
gender	5.521	0.019
start_travel_to_airport	17.426	0.002

**Table 7:** General results for ordered logit model for sustainable travel choices of passengers going to the airport.

Category	Wald Stat.	*p*-Value
Const	42.600	0.000
director/executive	10.872	0.001
manager	4.101	0.043
business owner	4.510	0.034
not employed	2.855	0.091
pensioner	6.058	0.014
assessing the duration	5.562	0.018
type of provider	11.694	0.001
place of living	5.576	0.018
start_travel_to_airport	31.810	0.000

**Table 8:** Detailed results for ordered logit model presented in Table 7.

Category	Level	Coeff	St. Err	Wald Stat.	Upper 95%	Lower 95%	*p*-Value
Const1		1.950	0.509	14.663	0.952	2.948	0.000
Const2		2.490	0.518	23.073	1.474	3.506	0.000
director/executive		3.423	1.038	10.872	1.388	5.458	0.001
manager		0.725	0.358	4.101	0.023	1.427	0.043
business owner		1.027	0.484	4.510	0.079	1.975	0.034
not employed		1.327	0.785	2.855	−0.212	2.866	0.091
pensioner		2.489	1.011	6.058	0.507	4.472	0.014
assessing the duration		−0.677	0.287	5.562	−1.240	−0.114	0.018
type of provider	LCC	−0.534	0.156	11.694	−0.841	−0.228	0.001
place of living	other	0.384	0.163	5.576	0.065	0.703	0.018
start_travel_to_airport *	*P*	−0.194	0.340	0.324	−0.860	0.473	0.569
start_travel_to_airport	M	−1.712	0.329	27.081	−2.357	−1.068	0.000
start_travel_to_airport	A	0.212	0.435	0.237	−0.641	1.064	0.626
start_travel_to_airport	R	2.193	0.826	7.047	0.574	3.811	0.008
Scale		1.000	0.000		1.000	1.000	

* M—Gdańsk, A—Tricity Agglomeration, R—Pomeranian Region, *P*—outside the Pomeranian Region, but in Poland.

## Data Availability

The data presented in this study are openly available in FigShare at DOI: 10.6084/m9.figshare.18018926.

## Appendix A

**Table A1:** Correlation matrix for the ordered logit (dependent variable: assessing the travel time).

	Const1	Const2	Business	Regular Employee	Time (Minutes)	Gender 1	Start_Travel_to_Airport 1	Start_Travel_to_Airport 2	Start_Travel_to_Airport 3	Start_Travel_to_Airport 4
Const1	1	0.792	−0.409	−0.288	−0.804	−0.027	0.004	−0.525	−0.338	−0.139
Const2	0.792	1	−0.331	−0.236	−0.722	−0.072	−0.038	−0.414	−0.274	−0.147
business	−0.409	−0.331	1	0.261	0.099	0.138	0.062	−0.139	−0.049	−0.078
regular employee	−0.288	−0.236	0.261	1	0.032	0.136	−0.058	−0.055	−0.042	−0.036
Time (minutes)	−0.804	−0.722	0.099	0.032	1	−0.021	−0.249	0.607	0.46	0.204
gender 1	−0.027	−0.072	0.138	0.136	−0.021	1	−0.028	−0.013	−0.015	−0.013
start_travel_to_airport 1	0.004	−0.038	0.062	−0.058	−0.249	−0.028	1	−0.142	−0.231	−0.151
start_travel_to_airport 2	−0.525	−0.414	−0.139	−0.055	0.607	−0.013	−0.142	1	0.195	0.032
start_travel_to_airport 3	−0.338	−0.274	−0.049	−0.042	0.46	−0.015	−0.231	0.195	1	−0.102
start_travel_to_airport 4	−0.139	−0.147	−0.078	−0.036	0.204	−0.013	−0.151	0.032	−0.102	1

**Table A2:** Correlation matrix for the ordered logit (dependent variable: sustainable transport mode).

	Const1	Const2	Director/Executive	Manager	Business Owner	Not Employed	Pensioner	Assessing the Duration	Type of Provider 1	Place of Living 1	Start_Travel_to_Airport 1	Start_Travel_to_Airport 2	Start_Travel_to_Airport 3	Start_Travel_to_Airport 4
Const1	1	0.98	−0.044	−0.187	−0.073	−0.063	0.082	−0.825	−0.233	0.087	−0.146	−0.632	−0.206	0.359
Const2	0.98	1	−0.033	−0.174	−0.063	−0.054	0.092	−0.821	−0.245	0.095	−0.143	−0.642	−0.199	0.36
director/executive	−0.044	−0.033	1	0.121	0.086	0.046	0.041	−0.003	0.055	0.077	0.02	−0.072	0.03	0.016
manager	−0.187	−0.174	0.121	1	0.215	0.107	0.09	0.022	0.158	0.083	0.053	−0.078	0.044	0.008
business owner	−0.073	−0.063	0.086	0.215	1	0.088	0.082	−0.058	0.048	0.089	0.057	−0.076	0.044	−0.003
not employed	−0.063	−0.054	0.046	0.107	0.088	1	0.072	0.032	−0.169	0.156	0.045	−0.072	0.008	0.039
pensioner	0.082	0.092	0.041	0.09	0.082	0.072	1	−0.122	−0.159	0.044	0.066	−0.179	0.008	0.02
assessing the duration	−0.825	−0.821	−0.003	0.022	−0.058	0.032	−0.122	1	0.112	−0.026	−0.191	0.325	0.084	−0.041
type of provider 1	−0.233	−0.245	0.055	0.158	0.048	−0.169	−0.159	0.112	1	−0.117	−0.095	0.26	0.051	−0.024
place of living 1	0.087	0.095	0.077	0.083	0.089	0.156	0.044	−0.026	−0.117	1	0.161	−0.051	0.21	0.077
start_travel_to_airport 1	−0.146	−0.143	0.02	0.053	0.057	0.045	0.066	−0.191	−0.095	0.161	1	0.331	0.129	−0.510
start_travel_to_airport 2	−0.632	−0.642	−0.072	−0.078	−0.076	−0.072	−0.179	0.325	0.26	−0.051	0.331	1	0.193	−0.546
start_travel_to_airport 3	−0.206	−0.199	0.03	0.044	0.044	0.008	0.008	0.084	0.051	0.21	0.129	0.193	1	−0.468
start_travel_to_airport 4	0.359	0.36	0.016	0.008	−0.003	0.039	0.02	−0.041	−0.024	0.077	−0.510	−0.546	−0.468	1
